# Digital analysis of unilateral ptosis repair: external levator advancement vs. Müller’s muscle conjunctival resection

**DOI:** 10.5935/0004-2749.2023-0028

**Published:** 2024-03-05

**Authors:** Serdar Bilici, Tomurcuk Harbigil-Sever, Suat Hayri Ugurbas

**Affiliations:** 1 Department of Ophthalmology, School of Medicine, Zonguldak Bulent Ecevit University, Zonguldak, Turkey; 2 Department of Ophthalmology, Van Training and Research Hospital, Van, Turkey

**Keywords:** Blepharoptosis, Eyelids, Conjunctiva, Oculomotor muscles, Image processing, computer-assisted, Treatment outcome

## Abstract

**Purpose:**

Evaluation of lid contour and marginal peak point changes to compare outcomes of external levator advancement and Miiller’s muscle conjunctival resection surgery in unilateral ptosis.

**Methods:**

We reviewed the charts of unilateral ptosis patients who underwent external levator advancement or Miiller’s muscle conjunctival resection. Eyelid contour analysis was conducted on preoperative and 6-month postoperative digital images. This was performed with the multiple margin reflex distances technique, measuring the vertical distance from a line intersecting the center of the pupil to the eyelid margin at 10 positions at 2 mm intervals. The marginal peak point changes were analyzed digitally using the coordinates of the peak point according to the pupil center. Each position’s mean distance was compared preoperatively, postoperatively, and with the fellow eyelid.

**Results:**

Sixteen patients underwent external levator advancement and 16 patients had Miiller’s muscle conjunctival resection. The mean margin reflex distance was improved by both techniques (1.46 *vs.* 2.43 mm and 1.12 *vs.* 2.25 mm, p=0.008 and p=0.0001 respectively) and approached that of the fellow eyelid (2.43 *vs.* 2.88 and 2.25 *vs.* 2.58 mm, p=0.23 and p=0.19, respectively). However, statistically significant lid margin elevation was limited to between the N6 and T6 points in the external levator advancement group. Whereas, significant elevation was achieved along the whole lid margin in the Miiller’s muscle conjunctival resection group. The marginal peak point was shifted slightly laterally in the external levator advancement group (p=0.11).

**Conclusions:**

Both techniques provide effective lid elevation, however, the external levator advancement’s effect lessens toward the canthi while Müller’s muscle conjunctival resection provides more uniform elevation across the lid margin. The margin reflex distance alone is not sufficient to reflect contour changes.

## INTRODUCTION

In addition to their functional roles, eyelids have a cosmetic role in compounding shape, height, contour, and especially symmetry^(1)^. Therefore, satisfactory ptosis treatment should ensure those compounds as much as possible. The margin reflex distance (MRD1) is often used alone in outcome studies to characterize the eyelid position after ptosis repair^(2,3,4)^. The upper eyelid contour is arched, however, MRD1 reflects only the central portion of the eyelid. Therefore, MRD1 alone cannot fully reflect the contour changes or show symmetry after eyelid surgery^(5)^.

Assessment of eyelid contour changes in ptosis repair have usually been qualitative with results classified as good, fair, and poor^(6,7)^. However, this is subjective and there is no standardization between centers or raters. With the widespread use of digital image analysis systems, several methods have been developed to evaluate ptosis surgery outcomes^(8,9,10,12)^. Danesh et al. proposed a new approach named multiple MRD1s to measure lid heights at 2 mm intervals, which enables objective symmetry comparisons of ptosis surgery techniques^(13)^.

In this study, the assessment of unilateral ptosis patients enabled comparison with the non-ptotic eyelid to assess surgical outcomes. We evaluated the lid contour changes and made symmetry analyses according to the non-ptotic fellow eyelids to compare the outcomes of external levator advancement (ELA) and Miiller’s muscle conjunctival resection (MMCR).

## METHODS

This study was approved by the Ethics Committee of Bulent Ecevit University and adhered to the tenets of the Declaration of Helsinki. In this retrospective, comparative, cohort study, two groups of subjects were compared. The first group comprised subjects who underwent ELA and the second group were patients who underwent MMCR for the management of involutional eyelid ptosis with levator function greater than 10 mm. Unilateral ptosis was defined as MRD1 ≤2.0 mm and more than a 1 mm difference in MRD1 between two eyelids^(14)^.

Patients were excluded if they were less than 18 years of age or had a diagnosis other than involutional ptosis, any eyelid pathology (e.g., ectropion, entropion, blepharospasm, or dermatochalasis covering the upper lid margin), enophthalmos or exophthalmos; also, if they had undergone bilateral surgery, upper eyelid blepharoplasty with MMCR or ELA, or external or internal browpexy. We also excluded patients with missing data, unsuitable photographic data, or who were not followed-up for least 6 months postoperatively.

All patients had a complete ophthalmologic examination before surgery. The MRD1, described as the distance between the pupillary light reflex and upper eyelid margin, and levator function, documented in millimeters, were recorded. Patients with desirable lid elevation after the instillation of 2.5% phenylephrine underwent MMCR, while those who did not respond to the phenylephrine test underwent ELA.

### Surgical technique

ELA was performed through a lid crease incision marked according to the fellow eyelid. The orbicularis muscle was dissected until the tarsal plate was defined. The preaponeurotic fat pad was exposed and the septum was opened. The dehisced levator aponeurosis was identified and sutured to the upper third of the tarsal plate with a double-armed 6/0 vicryl suture. After placing the patient in the sitting position, the eyelid height was evaluated. More sutures were applied as necessary to achieve the appropriate eyelid position. The lid crease was reformed and the skin closed with a 6/0 prolene suture.

In MMCR, the upper lid was turned over with the Desmarres retractor. Points five mm nasal and temporal to the steepest middle point of the upper edge of the tarsus were marked. A Putterman clamp was applied after marking half of the intended amount of resection of the conjunctiva and the Müller’s muscle. A 6/0 prolene suture was driven under the clamp running in both directions with the knot externalized at the lid crease. The clamped conjunctiva-Müller tissue was resected with a No.11 scalpel. The surgery was completed by ensuring hemostasis.

All procedures were performed under local anesthesia by the same surgeon (SHU) in Bulent Ecevit University Hospital between 2003 and 2010.

### Image analysis

Preoperative and 6th-month postoperative photographs were taken with the patient in the primary position with a digital camera positioned in the frontal plane at pupil height. All photographs were taken in the same room (in the oculoplastic department), with the same digital camera, under the same conditions of lighting and distance (1 m away from the patient), by the same person (SHU).

Digital image analysis was performed using ImageJ (National Institutes of Health, Bethesda, Maryland). For each image, a line was drawn between the patient’s lateral canthi and according to the slope of this line, the photographs were rotated to ensure the head was not tilted. Measurements were calibrated using a standard white-to-white diameter of 11.77 mm for men and 11.64 mm for women, as previously described^(15)^.

**Figure 1:**
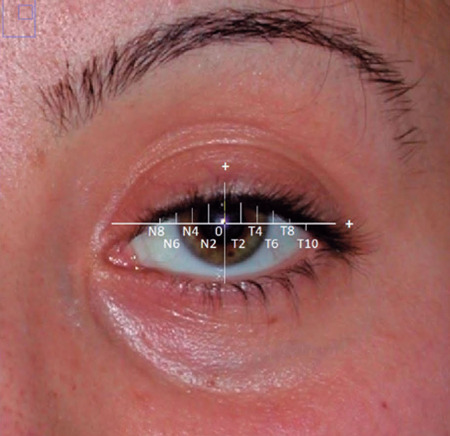
Multiple MRD1 measurement points. T: temporal to pupil center, N: nasal to pupil center.

The “multiple MRD1s” technique was used to analyze the lid contours for the pre- and postoperative photographs of all eyes^(13)^. The center of the pupil was marked manually according to its diameter. A horizontal line (with 0-degree of slope) was drawn and placed to pass through the center of the pupil. This horizontal line was marked out at 2 mm intervals, 8 mm medially, and 10 mm laterally, using the center of the pupil as a reference point (0,0). This created 10 reference points, including the center of the pupil, for each eye. From each of these points, a vertical line was drawn to the margin of the upper eyelid where the x coordinate represents the horizontal distance from the pupil center and the y coordinate represents the vertical height. The y coordinate at x=0 represents the MRD1 (Figure 1).

**Figure 2:**
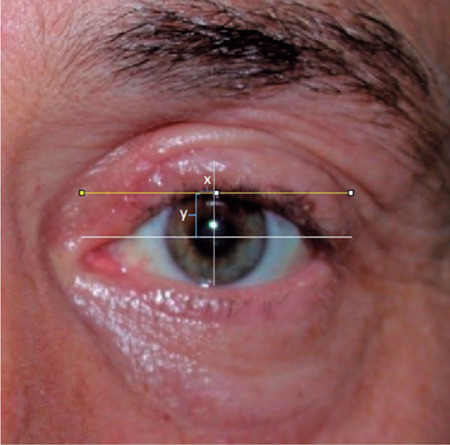
Lid margin peak point measurement. White horizontal line: mid-pupillary line, Yellow horizontal line: The line moving upward until the last point of contact with the margin represent the upper eyelid peak point; x is the horizontal distance of the peak point to the pupil center; y is the vertical distance of the peak point to the pupil center.

We used a previously described algorithm to determine the marginal peak point^(8)^. A horizontal line was drawn and moved upwards until it formed a tangent to the upper eyelid margin, the point of contact between the eyelid margin and line representing the upper eyelid peak. A vertical line was drawn through the pupil’s center to determine the y coordinate of the peak point. The horizontal distance between the peak point and the mid-pupillary line represented the x coordinate. By convention, negative values are assigned to marginal peaks located nasal to the mid-pupillary line and vice versa (Figure 2).

### Statistical analysis

Comparisons of preoperative and postoperative measurements were made. The changes in the y coordinates at each of the 10 points on the lid and the x coordinate of the peak point were calculated. Comparisons of x and y coordinate distances were made within groups with a t-test. Analyses were performed with IBM SPSS Statistics 22.0 (SPSS Inc, Chicago IL).

## RESULTS

Thirty-two patients with unilateral ptosis were enrolled in the study. The group comprised 22 male and 10 female patients, with ages ranging from 18 to 76 years (mean age: 49.7 years); 16 patients had ELA, while 16 patients had MMCR. The patient demographics are shown in table 1.

**Table 1:** Patient demographics and baseline margin reflex distances

	Total	ELA	MMCR
**No. of patients**	**32**	**16**	**16**
Age (mean ± STD, years)	49.7 ± 15.3	48.2 ± 15.7	51.3 ± 15.2
Gender			
Female	10	6	4
Male	22	10	12
Preoperative MRD1 (mean ± STD, mm)	1.29 ± 1.1	1.46 ± 1.45	1.12 ± 0.74
Fellow eyelid MRD1 (mean ± STD, mm)	2.73 ± 1.19	2.88 ± 1.69	2.58 ± 0.94

In the ELA and MMCR Groups, the mean MRD1 values of ptotic eyelids were 1.46 ± 1.45 mm and 1.12 ± 0.74 mm, respectively, preoperatively. Postoperatively, they were 2.43 ± 1.23 mm and 2.25 ± 0.72 mm, respectively. The mean MRD1 values of the non-ptotic fellow eyelids in the ELA and MMCR Groups were 2.88 ± 1.69 mm and 2.58 ± 0.94 mm, respectively. Both surgical techniques resulted in significant improvements in MRD1 values (p=0.008 and p=0.0001, respectively), with the postoperative values in both groups being close to those of the non-ptotic eyelids (p=0.23 and p=0.19, respectively).

**Figure 3:**
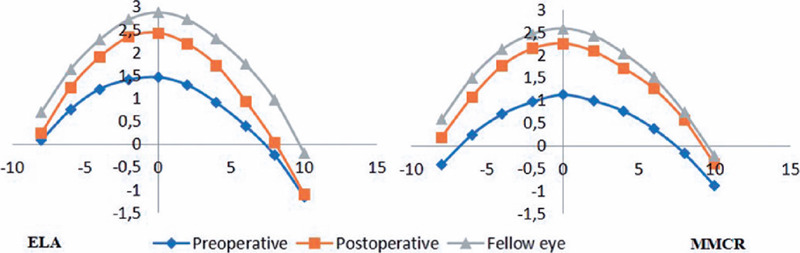
Multiple MRD1 changes and comparison with the fellow eye. ELA: External levator advancement, MMCR: Müllers muscle conjunctival resection.

The y coordinates at 10 points along the eyelid margin of pre- and postoperative ptotic eyelids and non-ptotic fellow eyelids in the ELA and MMCR Groups are summarized in figure 3 and table 2.

**Table 2:** Multiple MRD1 preoperative and postoperative positions of the ptotic and fellow eyelids

ELA Multiple MRD1s	N8	N6	N4	N2	0 (MRD1)	T2	T4	T6	T8	T10
Preoperative	0.08	0.75	1.20	1.40	1.46	1.29	0.90	0.39	–0.24	–1.14
	p=0.21	**p=0.01**	**p=0.003**	**p=0.005**	**p=0.008**	**p=0.01**	**p=0.01**	**p=0.03**	p=0.16	p=0.4
Postoperative	0.23	1.23	1.90	2.34	2.43	2.19	1.72	0.93	0.02	–1.0
	p=0.26	p=0.26	p=0.28	p=0.26	p=0.23	p=0.2	p=*0.18*	p=0.14	p=0.13	p=0.16
Fellow eye	0.61	1.64	2.31	2.82	2.88	2.78	2.51	1.90	1.06	–0.17

MMCR Multiple MRD1s	N8	N6	N4	N2	0 (MRD1)	T2	T4	T6	T8	T10
Preoperative	–0.42	0.24	0.69	0.96	1.12	0.99	0.76	0.37	–0.16	–0.87
	**p=0.008**	**p=0.0007**	**p<0.0001**	**p<0.0001**	**p<0.0001**	**p<0.0001**	**p<0.0001**	**p=0.0001**	**p=0.001**	**p=0.008**
Postoperative	0.18	1.07	1.76	2.14	2.25	2.08	1.71	1.25	0.57	–0.40
	p=0.19	p=0.16	p=0.17	p=0.20	p=0.19	p=0.2	p=0.22	p=0.29	p=0.36	p=0.35
Fellow eye	0.54	1.45	2.12	2.46	2.58	2.42	2.04	1.49	0.74	–0.19

Significant elevation limited to between N6 and T6 positions in the ELA Group while at all positions in the MMCR Group.

In the MMCR Group, the y coordinates at all 10 points had a significant elevation postoperatively and were similar to those of the non-ptotic fellow eyelids. In the ELA Group, statistically significant eyelid elevation was limited to the N6–T6 positions, with no significant changes at the N8, T8, and T10 positions (Table 2).

**Figure 4:**
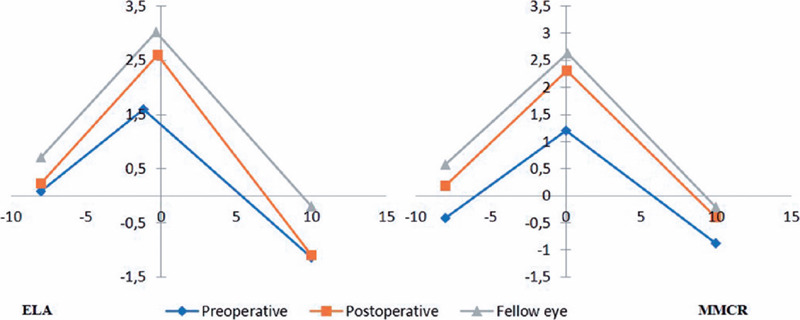
Lid margin peak point changes and comparison with the fellow eye. ELA: External levator advancement, MMCR: Müllers muscle conjunctival resection.

The marginal peak point x and y coordinates of the pre-and postoperative ptotic eyelids and non-ptotic fellow eyelids in the ELA and MMCR Groups are summarized in figure 4 and table 3.

The y coordinates of the marginal peak points of pre-and postoperative ptotic eyelids and non-ptotic fellow eyelids were 1.59 mm, 2.6 mm, and 3.01 mm in the ELA Group and 1.2, 2.31, and 2.62 in MMCR Group respectively. The y coordinates of the marginal peak points were changed significantly by both the ELA and MMCR techniques (p = 0.002 and p<0.001, respectively), and became significantly close to those of the non-ptotic fellow eyelids (p = 0.27 and p=0.2, respectively). The x coordinates of the marginal peak point of pre-and postoperative ptotic eyelids and non-ptotic fellow eyelids were –1.18 mm, –0.24 mm, and –0.32 mm respectively in ELA and 0.03, 0.07, and 0.09 mm respectively in the MMCR Group. In the ELA Group, the marginal peak point was more nasally placed in the preoperative ptotic eyelids than with the non-ptotic fellow eyelids (–1.18 vs. –0.32 mm), and a temporal shift in the x coordinate of the peak point was achieved by ELA surgery (from –1.18 to –0.24 mm). This shift, however, was not significantly important (p = 0.11). In contrast, the x coordinates of the marginal peak points in the MMCR Group were in a similar range. Both surgical techniques achieved similar postoperative x measurements in comparison to non-ptotic fellow eyelids (p = 0.36, p = 0.49, respectively) (Table 3).

## DISCUSSION

We investigated the eyelid contour changes achieved by ELA and MMCR in a unilateral ptosis cohort. Both ELA and MMCR provided a significant increase in MRD1 and achieved a similar profile to the non-ptotic fellow eyelid. Although the postoperative MRD1 in the ptotic and non-ptotic fellow eyelids were similar in both groups, elevation across the length of the eyelid decreased toward the canthi in ELA, while it was more uniform in MMCR. Statistically significant lid margin elevation was limited to the lid between N6 and T6 positions in the ELA Group, while it was observed at all 10 positions in the MMCR Group. The height of the lid margin peak point improved and approached that of the non-ptotic fellow eyelid in both groups. The lid margin peak point of ptotic eyelids was placed slightly nasal in the ELA Group preo-peratively and there was a postoperative temporal shift in the marginal peak point.

In clinical practice MRD1, palpebral fissure height and levator function are used to evaluate the outcomes of eyelid surgery. However, these measurements cannot account for the entire eyelid contour, peaks, and notching or reflect changes in lid contour deformities after surgery. They also differ according to the experience of the examiner^(16)^. After digital image analysis had been established, many groups attempted to quantify eyelid contour. Some studies used temporal and nasal area ratios, some used multiple radial midpupil lid distances and even third-degree equations of polynomial functions have been used to analyze eyelid contours^(8,9,10,11,12,17,18,19,20)^. In 2018, Danesh et al. described a new method with stable positions on the eyelid meridian for pre- and postoperative measurements. The measurements represent “multiple MRD1s”, are repeatable, and can easily be compared over time and between groups of patients^(13)^. Therefore, we used this method to analyze the lid contour changes in the two ptosis repair techniques.

We observed that MMCR provides more uniform elevation across the length of the eyelid than ELA does, although both techniques achieved similar MRD1 elevation. Danesh et al.^(13)^ also achieved less elevation of lid margin at the T10 and N8 positions with ELA than with MMCR, however, the difference was not statistically significant, as in our study. This result may have arisen because of the lower positioning preoperatively of the lid margins at T10 and N8 in both study’s ELA Groups. More likely, it is due to the different patterns of vectorial forces created by each surgical technique. If one thinks of ptosis surgery as a procedure to raise the top of a tent, ELA forms a bell tent because the main suture on the upper eyelid peak point provides a central support like a bell tent pole. In contrast, MMCR forms a dome tent with widespread support provided by the continuous suture line. With the same peak height, more height is gained over a wider area with the dome tent. The height reached in the center is not reflected in the edges to the same degree in the bell tent. Similarly, in comparison to MMCR, the more central force provided by ELA may not elevate the lid margins toward the canthi, while MMCR provides more uniform elevation.

It has been reported that the marginal peak point in ptotic eyelids moves temporally by the main hanging suture in ELA^(8,19,20,21)^. Due to the unilateral ptosis design of our study, we noticed that the peak point of the ptotic eyelid shifted nasally in the ELA Group achieving peak point symmetry with the contralateral eyelid. Conversely, there was no significant change in the x coordinate of the marginal peak point pre- or postoperatively in the MMCR Group. This may be because of the asymmetric separation of the levator aponeurosis, which has a steeper and wider bifurcation angle on the medial horn than on the lateral horn^(22,23,24)^. In cases of levator aponeurosis dehiscence from the anterior tarsus, the marginal peak point of the ptotic eyelid may shift nasally.

Our study has some limitations. One is the two-dimensional nature of the analysis method. A three-dimensional assessment of the contour may be more accurate. Additionally, manual determination of the center of the pupil is a major limitation that needs to be automated by software. Another limitation is the low number of cases, which is due to a low prevalence of unilateral ptosis. In addition, this study was retrospective, so the researchers could not investigate whether these digital analyses would be helpful in surgical procedures if applied preoperatively. Prospective studies should be conducted on the preoperative use of digital analyses.

In conclusion, both ELA and MMCR provide effective lid elevation, but their distribution patterns of vectorial force are different. The more central force from ELA results in less elevation toward the canthi, while the more diffuse force derived from MMCR provides more uniform elevation across the lid margin. There is a nasal shift at the marginal peak point of ptotic eyelids in the ELA Group, however lateralization by ELA serves to maintain symmetry with the contralateral eyelid. Although MRD1 reflects functional improvement well, digital evaluation methods should be used to analyze lid contours and objectively assess cosmetic outcomes of ptosis surgery.
